# Assessment of chronic disease self-management in patients with chronic heart failure based on the MCID of patient-reported outcomes by the multilevel model

**DOI:** 10.1186/s12872-021-01872-3

**Published:** 2021-01-30

**Authors:** Jing Tian, Jinghua Zhao, Qing Zhang, Jia Ren, Linai Han, Jing Li, Yanbo Zhang, Qinghua Han

**Affiliations:** 1 Department of Cardiology, The 1St Hospital of Shanxi Medical University, 85 South Jiefang Road, Taiyuan, 030001 Shanxi Province China; 2 Department of Health Statistics, School of Public Health, Shanxi Medical University, 56 South XinJian Road, Taiyuan, 030001 Shanxi Province China; 3 Shanxi Provincial Key Laboratory of Major Diseases Risk Assessment, 56 South XinJian Road, Taiyuan, 030001 Shanxi Province China

**Keywords:** Patient-reported outcome, Chronic heart failure, Chronic disease self-management, Multilevel model, Minimal clinically important difference

## Abstract

**Purpose:**

The minimal clinically important difference (MCID) of a patient-reported outcome (PRO) represents the threshold value of the change in the score for that PRO. It is deemed to have an important implication in clinical management. This study was performed to evaluate the clinical significance of chronic disease self-management (CDSM) for patients with chronic heart failure based on the MCID of the chronic heart failure—PRO measure (CHF-PROM).

**Methods:**

A multicenter, prospective cohort study of 555 patients with heart failure were enrolled from July 2018. Advice of CDSM was provided in written form at discharge to all patients. Information regarding CHF-PROM and CDSM were collected during follow-up. Multilevel models were applied to dynamically evaluate the effects of CDSM for CHF-PROM scores, as well as its physical and psychological domains. MCID changes of the PRO were introduced and compared with β values of CDSM obtained from the multi-level models to further evaluate the clinical significance. The STROBE checklist is shown in Additional file 1.

**Results:**

Scores for CHF-PROM improved significantly after discharge. The multilevel models showed that a regular schedule, avoidance of over-eating, a low-sodium diet and exercise increased scores on CHF-PROM. Compared with the MCID, avoidance of over-eating (12.39 vs. 9.75) and maintenance of a regular schedule often (10.98 vs. 9.75), and exercise almost every day (11.36 vs. 9.75) reached clinical significance for the overall summary. Avoidance of over-eating (5.88 vs. 4.79) and a regular schedule almost every day (4.96 vs. 4.79) reached clinical significance for the physical scores. Avoidance of over-eating half of the time (5.26 vs. 4.87) and a regular schedule almost every day (5.84 vs. 4.87) demonstrated clinical significance for the psychological scores.

**Conclusions:**

This study observed an association of avoidance of over-eating and maintenance of a regular schedule with the improvement of CHF-PROM. It provides further evidence for management of heart failure.

*Trial Registration*: Current Prospective Trials NCT02878811; registered August 25, 2016; https://clinicaltrials.gov/ct2/show/NCT02878811?term=NCT02878811&draw=2&rank=1.

## Introduction

Chronic heart failure (CHF) affects 1.5–2.0% of the adult population in developed countries [1] and 0.9% of the population aged 35–74 years in China [2]. CHF is the most severe stage of heart disease and has poor outcomes [3]. Therefore, close attention has been paid to evaluation and improvement of the endpoints of CHF.

Patient-reported outcomes (PROs) which reflect patient-centered quality of life are among the most crucial endpoints as recommended by the United States Food and Drug Administration, the International Association for Pharmaceutical Economics and Outcome Research, and the International Society for Quality of Life Research [4]. In recent years, researchers have begun to use PROs to evaluate the effects of intervention measures in patients with chronic diseases, including CHF [5]. Chronic disease self-management (CDSM), which is recommended by the European Society of Cardiology, can improve the outcomes of patients with heart failure [6]. However, the effects of CDSM on PROs are highly heterogeneous among these patients [7, 8].

Some deficiencies of previous studies may have interfered with the results. First, multi-point dynamic follow-up can accurately reflect the real-time changes in the disease. However, traditional prognostic analysis methods (e.g., logistic regression and Cox regression) are not suitable for these non-independent data. Second, evaluation of the effect of CDSM on patients with CHF has been mostly dependent upon the statistical significance [7, 8], the professional clinical sense has largely been ignored. The minimal clinically important difference (MCID) of PROs represents the threshold value of the clinical change in the score. The MCID is deemed to have an important implication in clinical management. Therefore, in the present study, we applied a multi-level model to analyze the roles of CDSM based on the MCID of PROs to obtain more reliable and meaningful evidence.

## Methods

### Participants

Patients from three medical centers in the Shanxi province of China were enrolled from July 2018 to December 2019 according to predefined inclusion and exclusion criteria. The inclusion criteria were: age ≥ 18 years; diagnosed with HF according to current guidelines [3]; New York Heart Association (NYHA) functional class II–IV; and receipt of HF therapy in the past month. Patients who experienced acute cardiovascular events except acute onset of CHF in the past 2 months, had a life expectancy of < 1 year, could not understand or complete the questionnaire due to language barriers or intellectual disabilities, and those who refused to participate in this project were excluded.

### Procedure and data collection

During hospitalization, information regarding baseline data, the self-administered questionnaire, and CHF-PROM scores were collected. The advice of CDSM was provided in written form to all of the participants at discharge. All participants were followed-up at 1, 3, and 6 months after discharge in face-to-face consultations or telephone follow-up to obtain information regarding the self-administered questionnaire and CHF-PROM scores [9]. To ensure quality, all questionnaires were administered by professionally trained individuals.

CDSM included medication use, a regular schedule, keeping warm, dietary instructions, health education, smoking cessation, temperance, and exercise. Dietary instructions included a low-sodium diet (LSD), a low-fat diet, and the avoidance of over-eating. Among these strategies, a regular schedule was defined as maintaining relatively fixed sleep and wake times, and an LSD as intaking < 5 g of salt per day.

Baseline information included patient’s age, sex, height, weight, marital status, education, annual income, family history of cardiovascular disease, NYHA functional class, blood pressure, and complications. The Charlson Comorbidity Index (CCI) was applied to assess complications [10].

The self-administered questionnaire was developed to assess CDSM. The questionnaire contained all strategies provided at discharge as mentioned above, with responses scored on a 5-point Likert, as follows: 0 (never happens); 1 (happens occasionally); 2 (happens half of the time); 3 (happens often); and 4 (happens every day).

The CHF-PROM developed by the authors’ research group was applied in this study. This questionnaire contains 57 items, 12 subdomains, and 4 domains, which consisted of physical, psychological, social, and therapeutic domains [9]. Patients responded to each item on a 5-point Likert scale to reflect how often they had experienced each issue during the past 2 weeks (0 = never, 1 = occasionally, 2 = about half of the time, 3 = often, and 4 = almost every day). All responses were transformed into scores based on the following principle: positively scored items were recorded as the original score plus 1, while negatively scored items were recorded as 5 minus the original score. After that, overall summary (OS), physical scores (PHYS) and psychological scores (PSYS) of CHF-PROM were calculated by adding scores of the corresponding items. Items were described as previously [9]. The structure of the CHF-PROs is shown in Additional file 2.

### Statistical analysis

Continuous variables are expressed as mean ± standard deviation (SD) or median (interquartile range). Cronbach’s α coefficient was applied to assess the data quality of the CHF-PROM. The variables that missing more than 15 percent were deleted. In addition, we added the data missing less than 15 percent with missForest. The backward method was used for statistically significant variables (*P* < 0.1). Univariate analysis of variables and calculation of MCID were performed using SPSS version 25.0 (IBM Corporation, Armonk, NY, USA). Further multilevel model assumptions were confirmed through analysis of residuals generated by MLwiN version 3.0 software (Centre for Multilevel Modelling, University of Bristol, Bristol, United Kingdom).

#### Multilevel model

The multilevel model, which can handle repeated measures data, was applied to assess the effect of CDSM strategies to the OS of CHF-PROM. The main concept of this model is to estimate variance at each level and consider the effect of the explanatory variables on the variance to estimate the regression coefficient effectively [11]. The model was constructed as follows:

1 $${Y}_{ij}={\beta }_{0j}+{\sum }_{i=1}{\beta }_{ij}{X}_{ij}+{e}_{ij}$$

2 $${\beta }_{0j}={\beta }_{0}+{u}_{0j}$$

3 $${\beta }_{ij}={\beta }_{j}+{u}_{ij}$$

${Y}_{ij}$ represents OS of CHFS-PROM taken from the $i$th person; ${e}_{ij}$ is the residual of the first level; ${\beta }_{0j}$ is the coefficient variable, which could be formulated by Eq. 2; ${\beta }_{0}$ and ${\beta }_{j}$ stand for fixed parameters representing the average of the intercept and slope, respectively; and ${u}_{0j}$ and ${u}_{ij}$ represent interindividual variability in intercepts and slopes via random effects. Maximum likelihood estimates can be computed from the covariance matrix.

#### Multivariate multilevel model

The multivariate multilevel model was fitted to assess CDSM strategies on PHYS, PSYS [11]. The multivariate variance components model was constructed as follows:

4 $${Y}_{itk}=\sum_{k}{D}_{k}({\beta }_{0ik}+{\beta }_{1ik}+{e}_{itk})$$

5 $${\beta }_{0ik}={\beta }_{0k}+{u}_{oik}$$

6 $${\beta }_{1ik}={\beta }_{1k}+{u}_{1ik}$$

In the equation above, ${Y}_{itk}$ represents the vector of two outcome measurements, taken from the $i$ th person at time $t$; ${D}_{k}$ is a pseudo variable, with a unique pseudo variable for each outcome; the $k$ response variable, ${\beta }_{0ik}$ is the overall intercept for person $i$; ${\beta }_{1ik}$ denotes a patient-specific slope; and ${e}_{itk}$ is residual error at time $t$ for person $i$.

In the present study, model 1 was the null model. Time was added to model 1 as an explanatory variable to establish model 2, which was used to study the effect of time on variables. Model 3 was constructed when baseline information and CDSM situation of participants were included in model 2.

#### MCID

Although *P* < 0.05 is often considered to be the criterion for evaluating the effectiveness of an intervention in PROs or QoL, the *P* value merely represents statistical significance. In our study, MCID was introduced to analyze its clinical significance to determine more effective CDSM strategies. ES of the distribution method was applied to calculate MCID according to characteristics of the current CHF-PROM data [12, 13]. ES was formulated as follows:

7 $$ES=\frac{{\stackrel{-}{x}}_{1}-{\stackrel{-}{x}}_{0}}{\sqrt{\sum {\left({x}_{0}-{\stackrel{-}{x}}_{0}\right)}^{2}/(n-1)}}$$

In the equation above, ${x}_{0}$ represents baseline scores of patients. ${\stackrel{-}{x}}_{0}$ represents the average baseline scores of individuals, and ${\stackrel{-}{x}}_{1}$ is the average follow-up scores of individuals. In our study, a moderate effect of 0.5 was used as the effect size statistic to estimate MCID.

Finally, *β* values of the multi-level model were compared with MCID. The first level of the variables was considered “0”, and multiplied the *β* value by the grade of levels minus “1”. The corresponding grade of variables up to MCID was defined as reaching clinical significance.

## Results

### Sample characteristics

Baseline characteristics of the patients are shown in Table 1. A total of 555 patients with CHF, with a mean ± SD age of 67.86 ± 14.58 years, was enrolled. Of these patients, 44.14% were female. 67.9% of them suffered from ischemic heart disease. Most participants were married (80.72%) and had a low level of education (below secondary high school [72.61%]), and 49.19% and 47.75% had a low and medium annual income, respectively.

**Table 1:** Baseline characteristics of patients with CHF

Variables	n = 555
Age	67.86 ± 14.58
Female	245 (44.14%)
*Marital state*	
Married	448 (80.72%)
Single	10 (1.80%)
Divorced/separated	9 (1.62%)
Widowed	88 (15.86%)
*Education*	
Illiteracy	63 (11.35%)
Low level	403 (72.61%)
Secondary school and high level	89 (16.04%)
*Income*	
Low	273 (49.19%)
Medium	265 (47.75%)
High	17 (3.06%)
Nonmanual workers	259 (46.67%)
Weight (kg)	66.67 ± 20.77
Height (cm)	165.27 ± 8.40
Systolic pressure (mmHg)	123.17 ± 19.61
Diastolic pressure (mmHg)	74.37 ± 13.00
Charslon score	2.47 ± 1.30
Ischemic heart disease	377 (67.9%)
Family history	115 (20.72%)
History of smoking	435 (78.38%)
History of drinking	468 (84.32%)
*NYHA*	
II	245 (44.15%)
III	211 (38.02%)
IV	99 (17.84%)
*Drugs*	
Nitrates	208 (37.48%)
Beta-blocker	378 (68.11%)
ACEI or ARB	250 (45.05%)
Aldosterone antagonist	359 (64.68%)
Diuretic	390 (70.27%)
Digoxin	113 (20.36%)

*ACEI* angiotensin converting enzyme inhibitors, *ARB* angiotensin receptor antagonist, *NYHA* New York Heart Association functional class

### CHF-PROM scores

Cronbach’s α coefficients for the physical domain, psychological domain, social domain, therapeutic domain, and overall scale were 0.893, 0.936, 0.835, 0.828, and 0.908, respectively. The mean CHF-PROM scores for OS, PSYS, and PHYS were 222.84 ± 23.18, 59.40 ± 10.84, and 89.60 ± 12.90, respectively. The scores were lowest during hospitalization, and improved significantly after discharge. The results are shown in Table 2.

**Table 2:** OS, PHYS and PSYS of CHF-PRO in different times

	Number	OS	PHY	PSY
Baseline	555	222.84 ± 23.18	59.40 ± 10.84	89.60 ± 12.90
One month	555	243.83 ± 14.84	69.88 ± 7.79	98.55 ± 6.14
Three months	501	245.46 ± 12.75	71.88 ± 6.59	100.21 ± 5.14
Six months	309	247.39 ± 12.61	72.49 ± 6.60	100.83 ± 4.74

*OS* overall scores, *PHYS* physical scores, *PSYS* psychological scores, *CHF-PRO* chronic heart failure-patient reported outcome

### Multilevel model of CDSM on CHF-PROM

**Fig.1:**
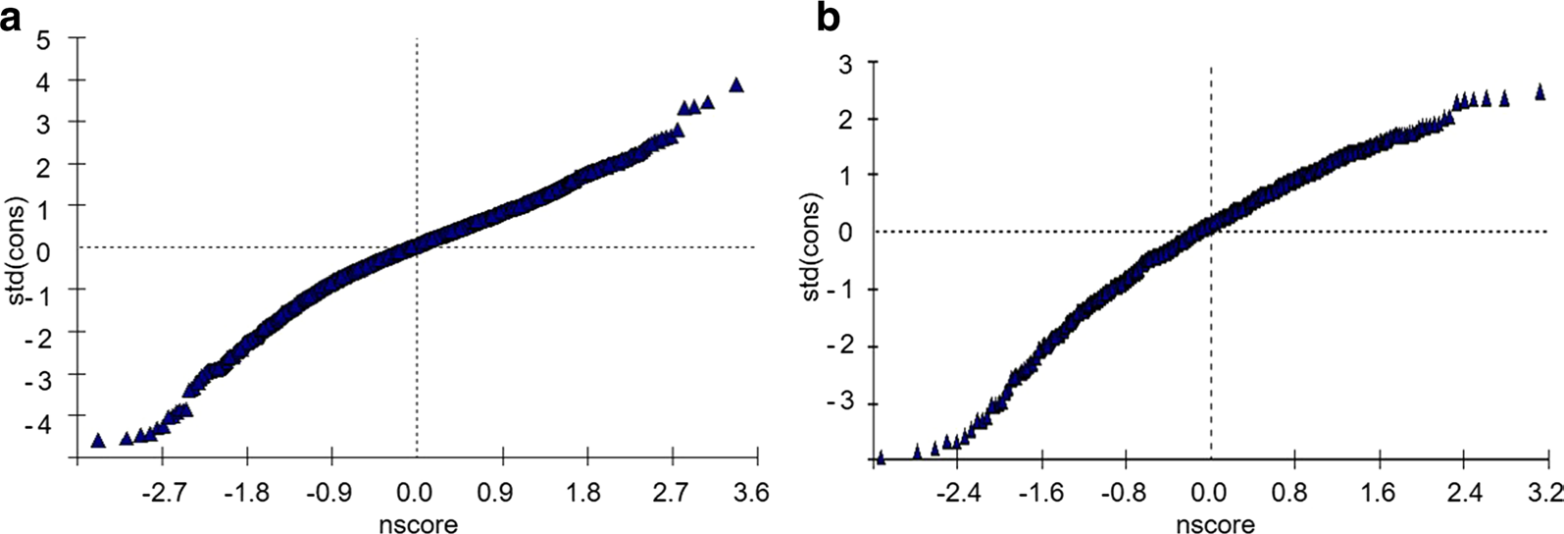
Residual normality test diagram of OS (**a**, **b**) are the residual normality test graphs of OS at the different time points level and the individual level, respectively. The ordinates of the diagrams represent the standardized residuals of each level, and the abscissas are their normal fractions. The curve of each figure represents the residual normality test of each level. The residual is normally distributed when the curve performs as a straight line

Three model levels were applied to assess CDSM strategies on OS of CHF-PROM; the results are summarized in Table 3. Model 1 demonstrated that the variance of level 2 (individual level) was statistically significant. It indicated that the data had aggregation and hierarchical structure at the individual level. Model 3 demonstrated that a regular schedule, avoidance of over-eating, an LSD and exercise improved OS in CHF-PROM. For each additional grade of the measures, OS increased by 3.66, 4.13, 1.71, and 2.84, respectively. Advanced age, female sex, and increased NYHA functional class were negatively correlated. A -2log likelihood was applied as the goodness fit evaluation index. The -2log likelihood of model 2 was smaller than model 1 (16,159.17 versus [vs.] 16,844.48); more specifically, the goodness fit of model 2 was better than model 1. For the same reason, model 3 had better goodness fit than model 2 (15,651.74 vs. 16,159.17). The residual distribution diagram is close to a straight line. Therefore, it indicated that the assumption of normal distribution of each level residuals was reasonable (Fig. 1).

**Table 3:** Three-level models for OS of CHF-PRO for patients with CHF

Parameters	Model 1	Model 2	Model 3
*Fixed effects*			
Intercept	238.22 (0.50)	218.60 (1.13)	207.40 (3.27)
Time		8.60 (0.36)*	4.58 (0.39)*
Age			− 0.17 (0.03)*
Female			− 1.66 (0.77)*
NYHA			− 4.15 (0.45)*
Regular schedule			3.66 (0.45)*
Avoid over-eating			4.13 (0.55)*
Low-sodium diet			1.71 (0.40)*
Exercises			2.84 (0.28)*
*Random effects*			
Level 2 (subjects)			
(Intercept)	41.20 (9.17)	384.07 (44.86)*	405.87 (40.26)*
(Time)		20.33 (4.61)*	27.46 (4.12)*
Level 1 (time point)			
(Intercept)	342.10 (13.04)	197.15 (9.05)*	146.74 (6.73)*
− 2 Log likelihood	16,844.48	16,159.17	15,651.74

OS, overall scores; CHF-PRO, chronic heart failure—patient reported outcome. NYHA, New York Heart Association functional class

**Fig. 2:**
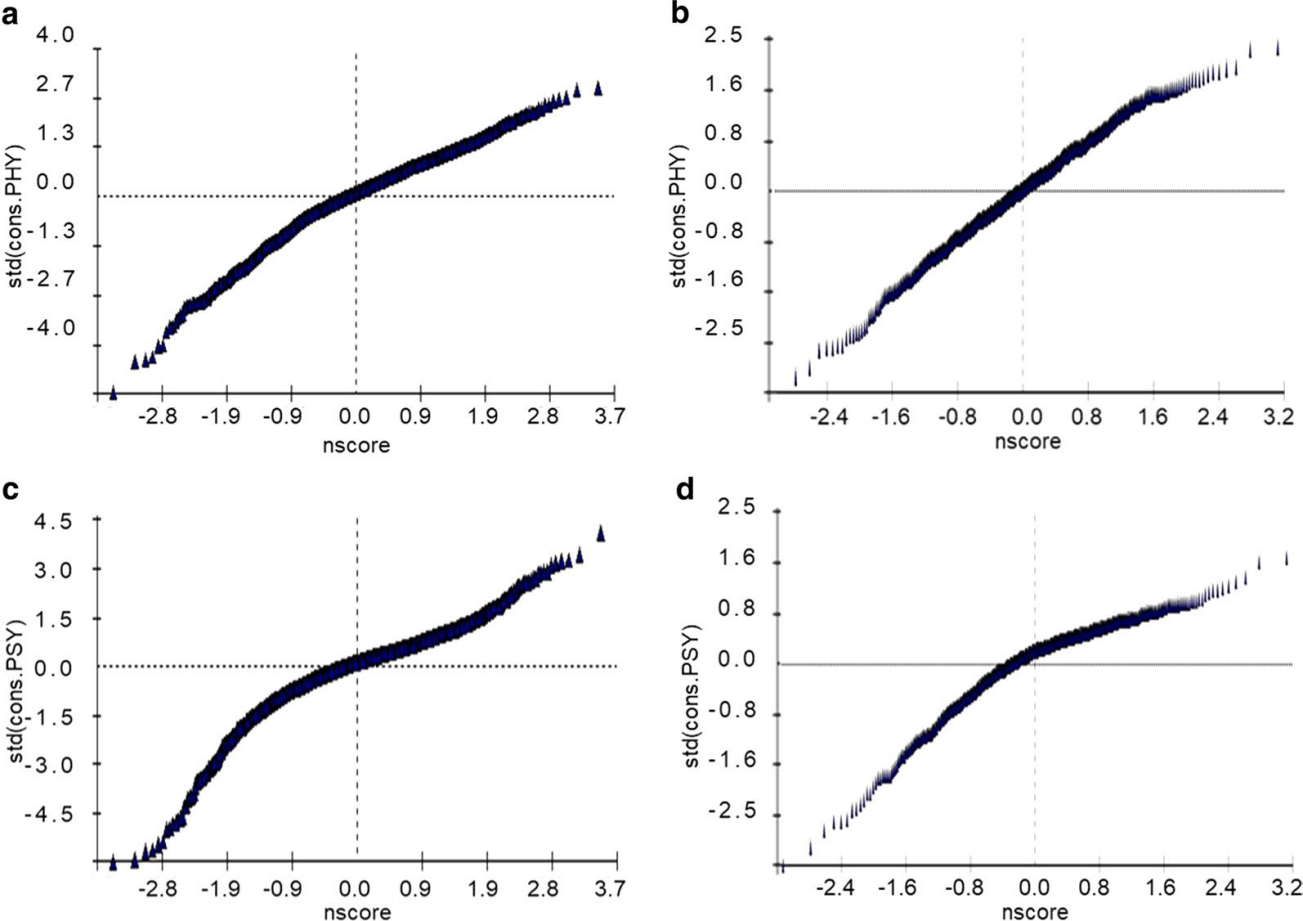
Residual normality test diagram of PHYS and PSYS (**a**, **b**) represent the residual normality tests of PHY at the timepoint level and the individual level, respectively. **c**, **d** Represent the residual normality test of PSY at the time-point level and the individual level, respectively

A two-variable, three-level model was applied to analyze the roles of CDSM strategies to PHYS and PSYS, the results are presented in Table 4. Model 1 demonstrated that the variance of level 3 (individual level) was statistically significant. It indicated that the data had aggregation and hierarchical structure at the individual level. According to the model, a regular schedule, avoidance of over-eating, an LSD and exercise increased PHYS and PSYS. For each additional grade of the measures, PHYS increased by 1.24, 1.96, 0.86, and 1.18 and PSYS increased by 1.46, 2.63, 0.76, and 0.55. In addition, taking angiotensin-converting enzyme inhibitors or angiotensin receptor blocker decreased the PSYS of patients with CHF. Advanced age, female sex, increased NYHA functional class and CCI were negatively correlated with PHYS and PSYS. A -2log likelihood demonstrated that the goodness fit of model 2 was better than model 1 (26,155.51 vs*.* 27,286.37), and model 3 was better than model 2 (25,458.50 vs. 26,155.51). The residual distribution diagram is close to a straight line. Therefore, it indicated that the assumption of normal distribution of each level residuals was reasonable (Fig. 2).

**Table 4:** Multilevel multivariate models for PHYS and PSYS of CHF-PRO

Parameters	Model 1		Model 2		Model 3	
	PHY	PSY	PHY	PSY	PHY	PSY
*Fixed effects*						
Intercept	68.18 (0.26)	97.02 (0.25)	58.68 (0.51)	88.43 (0.64)	63.04 (1.74)	77.73 (1.77)
Time			4.16 (0.17)*	3.71 (0.19)*	2.27 (0.18)*	2.15 (0.21)*
Age					− 0.12 (0.01)*	− 0.02 (0.01)*
Female					− 1.05 (0.40)*	− 1.36 (0.36)*
NYHA					− 3.10 (0.23)*	− 0.76 (0.22)*
CCI					− 0.43 (0.16)*	− 0.30 (0.14)*
ACEI or ARB					− 0.42 (0.45)	− 1.00 (0.40)*
Regular schedule					1.24 (0.22)*	1.46 (0.23)*
Low sodium diet					0.86 (0.19)*	0.76 (0.20)*
Avoid over-eating					1.96 (0.26)*	2.63 (0.28)*
Exercises					1.18 (0.14)*	0.55 (0.14)*
*Random effects*						
Level 3 (subjects)						
(Intercept)		13.85 (2.35)		67.50 (9.42)*		68.25 (7.96)*
(Intercept)		1.81 (1.85)		38.57 (8.90)*		49.07 (8.02)*
(Intercept)		10.50 (2.29)		148.65 (13.84)*		159.18 (13.40)*
(Time)				2.91 (1.01)*		4.47 (0.87)*
(Time)				8.58 (1.27)*		10.90 (1.25)*
Level 2 (time point)						
(Intercept)		78.32 (2.99)		47.40 (2.19)*		34.55 (1.60)*
(Intercept)		54.76 (2.65)		28.19 (1.77)*		17.02 (1.28)*
(Intercept)		85.00 (3.24)		46.88 (2.12)*		36.62 (1.67)*
− 2 Log likelihood		27,286.37		26,155.51		25,458.50

*ACEI* angiotensin converting enzyme inhibitors, *ARB* angiotensin receptor antagonist, *CCI* Charlson Comorbidity Index *CHF-PRO* chronic heart failure—patient reported outcome, *NYHA* New York Heart Association functional class, *PHYS* physical scores, *PSYS* psychological scores

### MCID and its interpretation to the multilevel model

The MCIDs for the scores of each dimension and domain and the total scale are shown in Table 5. The MCIDs for OS, PHYS, and PSYS were 9.75, 4.79, and 4.87, respectively. This indicates that scores for the CHF-PROM, physical domain, and psychological domain that changed by at least 9.75, 4.79, and 4.87 points were considered clinically significant.

**Table 5:** MCID of CHF-PRO

Field	Dimension	MCID		
		Dimension	Domain	Total of scale
Physical domain	Somatic symptoms…	2.46	4.79	9.75
	Appetite symptoms…	1.43		
	independence	2.35		
Psychological domain	Anxiety	2.63	4.87	
	Depression	1.56		
	Fear	0.71		
	Paranoia	0.69		
Social domain	Social support	1.63	2.14	
	Support utilization	1.26		
Therapeutic domain	Compliance	0.61	2.40	
	Satisfaction	2.00		
	Side effects of drugs	0.37		

*CHF-PRO* chronic heart failure—patient reported outcome, *MCID* minimal clinically important difference

**Fig. 3:**
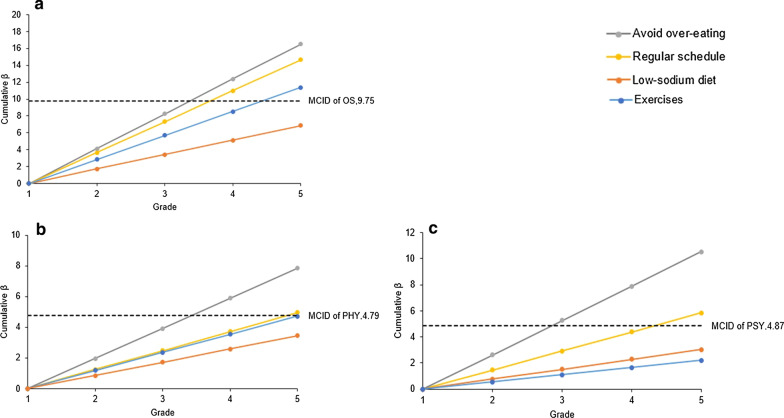
Comparation of MCID to the cumulative β for variables each point represents the value that the correspond β of strategy multiplied by (grade-1). MCID is shown as a dotted black line. The strategy is of the clinical significance when the its value is larger than MCID. **a** Represents the influence of management strategies on OS. **b**, **c** represent the influence of management strategies on PHY and PSY, respectively

Compared with MCID, the avoidance of over-eating of grade 4 and 5, regular schedule of grade 4 and 5 and exercise of grade 5 reached clinical significance for OS. Avoidance of over-eating of grade 4 and 5 and a regular schedule of grade 5 reached clinically significance for PHY. Regarding the PSY, avoidance of over-eating of grade 3, 4 and 5 and a regular schedule of grade 5 also demonstrated the clinical significance (Fig. 3).

## Discussion

The present study assessed the impact of several types of CDSM strategies on CHF-PROM scores. Here, we confirmed that maintenance of a regular schedule, avoidance of over-eating, an LSD and exercise could improve CHF-PROM scores in patients with CHF. Among these, however, only a regular schedule and avoidance of over-eating reached clinical significance based on the MCID of CHF-PROM. Compared to previous studies, various strategies were considered and changes in these over time were assessed. Moreover, based on statistical significance, clinical significance was emphasized by virtue of the MCID.

The characteristics of patients with CHF have an impact on PROs. In our study, a high NYHA functional class, female sex, and advanced age decreased the OS in CHF-PROM, as well as PHYS and PSYS. Moreover, CCI was negatively correlated with PHYS and PSYS. These factors have already been shown as influence factors of PROs in patients with CHF in previous studies [10, 14–16]. We used multivariate statistical methods to avoid the influence of these covariates on the results; thus, we were able to obtain CDSM strategies that improved CHF-PROM more accurately.

Results of our study demonstrated that maintaining a regular schedule improved CHF-PROM. The same result was obtained in previous studies using other PRO scales of HF. Broström et al. found that sleep disturbances affected virtually all dimensions of the Short-form 36 and Kansas City Cardiomyopathy Questionnaire (KCCQ) for patients with CHF, while daytime sleepiness decreased total Minnesota Living with Heart Failure (MLwHF) scores, as well as scores on physical and emotional subscales [17]. Liu et al. reported that poor sleepers had significantly lower scores in physical, psychological, and social domains of the World Health Organization Quality of Life-BREF (WHOQOL-BREF) scale [18]. Sleep disorders in patients with CHF are caused by sleep-disordered breathing, depression, and HF symptoms such as dyspnea and dysrhythmias [19]. These investigations were cross-sectional studies, and dynamic changes in sleeping habits and PROs were not observed. Our study applied a multilevel model to introduce time as a variable. A prospective cohort study using one-way repeated measures analysis reported that exercise and cognitive behavioral therapy may improve sleep quality and QoL in patients with CHF [20]. In our study, patients were informed that they should maintain a regular routine, regardless of the strategy they used. The results of our study emphasize the importance of a regular schedule in patients with CHF. Moreover, only patients who maintained a regular schedule virtually every day achieved MCID, reflecting that it is necessary for patients to be compliant with physician recommendations.

Over-eating often relies on patient perception and lacks objective indicators for evaluation. As such, few studies have extensively investigated this factor. Our study unexpectedly found that avoidance of over-eating dramatically decreased OS, as well as PHYS and PSYS in CHF-PROM. Research presented at the American Heart Association meeting in 2000 found that a single large meal led to a fourfold increase in heart attacks within 2 h of the meal [21]. A rich diet burdens the heart due to diversion of the circulation to the gastrointestinal tract following a meal. Such a diversion increases cardiac blood and causes further stress on the heart. Moreover, acute fluctuations in blood pressure and heart rate occur after a rich meal and lead to further damage to the heart [22]. If an individual with CHF consumed a large, high-salt meal, acute decompensation could even occur [23]. The avoidance of over-eating may improve CHF-PROM by decreasing the incidence of these types of adverse events. This result provides new evidence supporting the management of CHF and direction for future studies.

An LSD was recommended by the 2016 European Society of Cardiology Guidelines for CHF [3]. In the present study, we confirmed that an LSD increased OS, PHYS, and PSYS of CHF-PROM. Previous studies and the ongoing Geriatric Out-of-Hospital Randomized Meal Trial in Heart Failure (GOURMET-HF) study applied the KCCQ summary score as an indicator of QoL outcome and drew the same conclusion as that in our study [24–26]. Regarding PHYS, the reason for the increase may be that an LSD improved symptoms and signs of CHF [27, 28] and promoted exercise tolerance in patients [28]. However, few studies have focused on the relationship between LSD and psychological states. More studies are needed to confirm this and the mechanism also remains to be further elucidated. Adherence to an LSD has also been noted by researchers. Chung et al. confirmed that patients who adhered to an LSD perceived more benefits than those who were non-adherent [29]. All of the research above focused exclusively on statistical significance and ignored clinical significance. When MCID was introduced, it did not reach clinical significance, regardless of a patient's adherence to an LSD in this study. This also may be because some patients did not accurately calculate the amount of salt they ate at home. More stringent studies and investigations examining clinical significance are needed in the future.

Regular aerobic exercise is encouraged in patients with HF to improve functional capacity and symptoms, as per guideline recommendations [3]. Studies have shown that exercise can reduce all-cause mortality and readmission for patients with CHF; however, the effects of exercise on QoL remain uncertain [30]. A recent meta-analysis confirmed that exercise improved both exercise capacity and QoL compared with the no-exercise control group at the 12-month follow-up, but with weaker evidence for a treatment effect at the 6-month follow-up [31]. Our study demonstrated that exercise improved CHF-PROM. This is consistent with previous studies and provides the new evidence for the effect at the 6-month follow-up.

The findings of this study should be interpreted in light of its limitations. First, all advice adopted in this study was beneficial to strategies for patients with CHF. Based on ethical considerations, we provided all participants with advice when they were discharged; as such, there was no control group. It revealed that the causal effect was not as strong as that from a randomized controlled trial. We will use randomized controlled trial design in future research to assess one of the meaningful strategies in this study. Second, although this was a multicenter study, all patients were from the Shanxi Province of China and, as such, the findings may be regionally biased. Larger-scale studies are needed in the future to confirm the findings in this regard. Finally, some of the CDSM strategies used in this study were not precisely defined. For example, a regular schedule did not limit the sleep time per day or apply related scales to measure sleep quality, which may have led to some imprecision. In future studies, we will further quantify the strategies addressed in this study to obtain more effective CDSM strategies for patients with CHF.

## Conclusions

This study observed an association of avoidance of over-eating and maintenance of a regular schedule with the improvement of CHF-PROM. Among them, only the strategies happened often or every day had the clinical significance. It prompts patients and physicians to give preference to certain strategies and enables them to understand more intuitively and profoundly the meaning of measure compliance.

## Supplementary Information

**Additional file 1:** STROBE Statement-Checklist of items.

**Additional file 2:** Structure of the CHF-PROs.

## Data Availability

The datasets used and/or analyzed during the current study are available from the corresponding author on reasonable request.

## Abbreviations

CCI: Charlson Comorbidity Index
CDSM: Chronic disease self-management
CHF: Chronic heart failure
CHF-PROM: Chronic heart failure—patient-reported outcome measure
HF: Heart failure
KCCQ: Kansas City Cardiomyopathy Questionnaire
LSD: Low-sodium diet
MCID: Minimal clinically important difference
NYHA: New York Heart Association
OS: Overall summary
PHYS: Physical scores
PRO: Patient-reported outcome
PSYS: Psychological scores
QoL: Quality of life
SD: Standard deviation

## References

[1] Yancy CW, Jessup M, Bozkurt B, Butler J, Casey DE, Colvin MM (2017). 2017 ACC/AHA/HFSA focused update of the 2013 ACCF/AHA guideline for the management of heart failure: a report of the American College of Cardiology/American Heart Association Task Force on Clinical Practice Guidelines and the Heart Failure Society of America. Circulation.

[2] Hu S, Gao R, Liu L, Zhu M, Wang W, Wang Y (2019). Summary of the 2018 Report on Cardiovascular Diseases in China. Chin Circ J.

[3] Ponikowski P, Voors AA, Anker SD, Bueno H, Cleland JGF, Coats AJS (2016). 2016 ESC Guidelines for the diagnosis and treatment of acute and chronic heart failure: The Task Force for the diagnosis and treatment of acute and chronic heart failure of the European Society of Cardiology (ESC) Developed with the special contribution of the Heart Failure Association (HFA) of the ESC. Eur Heart J.

[4] U.S. Department of Health and Human Services FDA Center for drug evaluation and research, U.S. Department of Health and Human Services FDA Center for biologics evaluation and research, U.S. Department of Health and Human Services FDA Center for devices and radiological health. Guidance for industry: patient-reported outcome measures: use in medical product development to support labeling claims: draft guidance. Health Qual Life Outcomes. 2006; 4(79): e1–e20. 10.1186/1477-7525-4-79 PMC1629006 17034633

[5] Kelkar AA, Spertus J, Pang P, Pierson RF, Cody RJ, Pina IL (2016). Utility of patient-reported outcome instruments in heart failure. JACC Heart Fail.

[6] Seferovic PM, Ponikowski P, Anker SD, Bauersachs J, Chioncel O, Cleland JGF, et al. Clinical practice update on heart failure 2019: pharmacotherapy, procedres, devices and patient management. An expert consensus meeting report of the Heart Failure Association of the European Society of Cardiology. Eur. J. Heart Fail. 2019; 21(10): 1–17. 10.1002/ejhf.1531 31129923

[7] Jonkman NH, Westland H, Groenwold RH, Agren S, Atienza F, Blue L (2016). Do self-management interventions work in patients with heart failure? An individual patient data meta-analysis. Circulation.

[8] Riegel B, Lee CS, Dickson VV, Medscape. Self care in patients with chronic heart failure. Nat Rev Cardiol. 2011; 8(11): 644–654. 10.1038/nrcardio.2011.95 21769111

[9] Tian J, Xue J, Hu X, Han Q, Zhang Y (2018). CHF-PROM: validation of a patient-reported outcome measure for patients with chronic heart failure. Health Qual Life Outcomes.

[10] Blinderman CD, Homel P, Billings JA, Portenoy RK, Tennstedt SL (2008). Symptom distress and quality of life in patients with advanced congestive heart failure. J Pain Symptom Manag.

[11] Goldstein H (2003). Multilevel statistical models.

[12] Sedaghat AR (2019). Understanding the Minimal Clinically Important Difference (MCID) of Patient-Reported Outcome Measures. Otolaryngol Head Neck Surg.

[13] Wright A, Hannon J, Hegedus EJ, Kavchak AE (2012). Clinimetrics corner: a closer look at the minimal clinically important difference (MCID). J Man Manip Ther.

[14] Carson P, Tam SW, Ghali JK, Archambault WT, Taylor A, Cohn JN (2009). Relationship of quality of life scores with baseline characteristics and outcomes in the African-American heart failure trial. J Card Fail.

[15] Lewis EF, Kim HY, Claggett B, Spertus J, Heitner JF, Assmann SF (2016). Impact of Spironolactone on Longitudinal Changes in Health-Related Quality of Life in the Treatment of Preserved Cardiac Function Heart Failure With an Aldosterone Antagonist Trial. Circ Heart Fail.

[16] Napier R, McNulty SE, Eton DT, Redfield MM, AbouEzzeddine O, Dunlay SM (2018). Comparing measures to assess health-related quality of life in heart failure with preserved ejection fraction. JACC Heart Fail.

[17] Broström A, Strömberg A, Dahlström U, Fridlund B (2004). Sleep difficulties, daytime sleepiness, and health-related quality of life in patients with chronic heart failure. J Cardiovasc Nurs.

[18] Liu JC, Hung HL, Shyu YK, Tsai PS (2011). The impact of sleep quality and daytime sleepiness on global quality of life in community-dwelling patients with heart failure. J Cardiovasc Nurs.

[19] F Johansson P, Dahlström U, Broström A. Factors and interventions influencing health-related quality of life in patients with heart failure: a review of the literature. Eur J Cardiovasc Nurs. 2006; 5(1): 5–15. 10.1016/j.ejcnurse.2005.04.011 15967727

[20] Chang YL, Chiou AF, Cheng SM, Lin KC (2016). Tailored educational supportive care programme on sleep quality and psychological distress in patients with heart failure: a randomised controlled trial. Int J Nurs Stud.

[21] American Heart Association (2000) ‘Heavy Meals May Trigger Heart Attacks.’ Science Daily.

[22] Cox HS, Kaye DM, Thompson JM, Turner AG, Jennings GL, Itsiopoulos C (1995). Regional sympathetic nervous activation after a large meal in humans. Clin Sci (Lond).

[23] Bibbins-Domingo K, Chertow GM, Coxson PG, Moran A, Lightwood JM, Pletcher MJ (2010). Projected effect of dietary salt reductions on future cardiovascular disease. N Engl J Med.

[24] Chen YW, Wang CY, Lai YH, Liao YC, Wen YK, Chang ST (2018). Home-based cardiac rehabilitation improves quality of life, aerobic capacity, and readmission rates in patients with chronic heart failure. Medicine (Baltimore).

[25] Colin-Ramirez E, McAlister FA, Zheng Y, Sharma S, Armstrong PW, Ezekowitz JA. The long-term effects of dietary sodium restriction on clinical outcomes in patients with heart failure. The SODIUM-HF (Study of Dietary Intervention Under 100 mmol in Heart Failure): a pilot study. Am. Heart J. 2015; 169(2): 274–281. 10.1016/j.ahj.2014.11.013 25641537

[26] Hummel SL, Karmally W, Gillespie BW, Helmke S, Teruya S, Wells J (2018). Home-Delivered Meals Postdischarge From Heart Failure Hospitalization. Circ Heart Fail.

[27] Philipson H, Ekman I, Forslund HB, Swedberg K, Schaufelberger M (2013). Salt and fluid restriction is effective in patients with chronic heart failure. Eur J Heart Fail.

[28] Welsh D, Lennie TA, Marcinek R, Biddle MJ, Abshire D, Bentley B (2013). Low-sodium diet self-management intervention in heart failure: pilot study results. Eur J Cardiovasc Nurs.

[29] Chung ML, Park L, Frazier SK, Lennie TA (2017). Long-term adherence to low-sodium diet in patients with heart failure. West J Nurs Res.

[30] Taylor RS, Walker S, Smart NA, Piepoli MF, Warren FC, Ciani O (2018). Impact of exercise-based cardiac rehabilitation in patients with heart failure (ExTraMATCH II) on mortality and hospitalisation: an individual patient data meta-analysis of randomised trials. Eur J Heart Fail.

[31] Taylor RS, Walker S, Smart NA, Piepoli MF, Warren FC, Ciani O (2019). Impact of exercise rehabilitation on exercise capacity and quality-of-life in heart failure: individual participant meta-analysis. J Am Coll Cardiol.

