# Supplementary material for: Assessment of chronic disease self-management in patients with chronic heart failure based on the MCID of patient-reported outcomes by the multilevel model
Source: BMC Cardiovasc Disord. 2021 Jan 30;21:58. doi: 10.1186/s12872-021-01872-3 (PMC7847136; doi:10.1186/s12872-021-01872-3)
Supplement: Supplementary file 1 — Additional file 1: STROBE Statement-Checklist of items. [file 12872_2021_1872_MOESM1_ESM.doc]

Supplementary 1 STROBE Statement-Checklist of items

|  | Item No | Recommendation |  | Page No. |
| --- | --- | --- | --- | --- |
| **Title and abstract** | 1 | (*a*) Influence of self-management on patient-reported outcomes for individuals with chronic heart failure: a multilevel model approach from a longitudinal study | √ | 1 |
|  |  | (*b*) Provide in the abstract an informative and balanced summary of what was done and what was found | √ | 2 |
| Introduction | | |  |  |
| Background/rationale | 2 | Explain the scientific background and rationale for the investigation being reported | √ | 3 |
| Objectives | 3 | State specific objectives, including any prespecified hypotheses | √ | 3 |
| Methods | | |  |  |
| Study design | 4 | Present key elements of study design early in the paper | √ | 4 |
| Setting | 5 | Describe the setting, locations, and relevant dates, including periods of recruitment, exposure, follow-up, and data collection | √ | 4-5 |
| Participants | 6 | (*a*) Give the eligibility criteria, and the sources and methods of selection of participants | √ | 4 |
| Variables | 7 | Clearly define all outcomes, exposures, predictors, potential confounders, and effect modifiers. Give diagnostic criteria, if applicable | √ | 4-5 |
| Data sources/ measurement | 8* | For each variable of interest, give sources of data and details of methods of assessment (measurement). Describe comparability of assessment methods if there is more than one group | √ | 4-5 |
| Bias | 9 | Describe any efforts to address potential sources of bias | N/A | N/A |
| Study size | 10 | Explain how the study size was arrived at | √ | 7 |
| Quantitative variables | 11 | Explain how quantitative variables were handled in the analyses. If applicable, describe which groupings were chosen and why | √ | 5 |
| Statistical methods | 12 | (*a*) Describe all statistical methods, including those used to control for confounding | √ | 5-7 |
|  |  | (*b*) Describe any methods used to examine subgroups and interactions | N/A | N/A |
|  |  | (*c*) Explain how missing data were addressed | √ | 5 |
|  |  | (*d*) If applicable, describe analytical methods taking account of sampling strategy | NA | NA |
|  |  | (e) Describe any sensitivity analyses | NA | NA |
| Results | | |  |  |
| Participants | 13* | (a) Report numbers of individuals at each stage of study—eg numbers potentially eligible, examined for eligibility, confirmed eligible, included in the study, completing follow-up, and analysed | √ | Table 2 |
|  |  | (b) Give reasons for non-participation at each stage | NA | NA |
|  |  | (c) Consider use of a flow diagram | NA | NA |
| Descriptive data | 14* | (a) Give characteristics of study participants (eg demographic, clinical, social) and information on exposures and potential confounders | √ | 8 |
|  |  | (b) Indicate number of participants with missing data for each variable of interest | NA | NA |
| Outcome data | 15* | Report numbers of outcome events or summary measures | NA | NA |
| Main results | 16 | (*a*) Give unadjusted estimates and, if applicable, confounder-adjusted estimates and their precision (eg, 95% confidence interval). Make clear which confounders were adjusted for and why they were included | √ | 8-10 |
|  |  | (*b*) Report category boundaries when continuous variables were categorized | NA | NA |
|  |  | (*c*) If relevant, consider translating estimates of relative risk into absolute risk for a meaningful time period | NA | NA |
| Other analyses | 17 | Report other analyses done—eg analyses of subgroups and interactions, and sensitivity analyses | NA | NA |
| Discussion | | |  |  |
| Key results | 18 | Summarise key results with reference to study objectives | √ | 10-12 |
| Limitations | 19 | Discuss limitations of the study, taking into account sources of potential bias or imprecision. Discuss both direction and magnitude of any potential bias | √ | 12 |
| Interpretation | 20 | Give a cautious overall interpretation of results considering objectives, limitations, multiplicity of analyses, results from similar studies, and other relevant evidence | √ | 13 |
| Generalisability | 21 | Discuss the generalisability (external validity) of the study results | √ | 10-12 |
| Other information | | |  |  |
| Funding | 22 | Give the source of funding and the role of the funders for the present study and, if applicable, for the original study on which the present article is based | √ | 14 |
