# Supplementary material for: Assessment of chronic disease self-management in patients with chronic heart failure based on the MCID of patient-reported outcomes by the multilevel model
Source: BMC Cardiovasc Disord. 2021 Jan 30;21:58. doi: 10.1186/s12872-021-01872-3 (PMC7847136; doi:10.1186/s12872-021-01872-3)
Supplement: Supplementary file 2 — Additional file 2: Structure of the CHF-PROs. [file 12872_2021_1872_MOESM2_ESM.docx]

Supplementary 2 Structure of the CHF-PROs

| Domain | Subdomain | Item |
| --- | --- | --- |
| Physical domain | somatic symptoms | PHD1-、PHD2-、PHD3-、PHD4-、PHD5-、PHD6-、PHD7-、PHD8- |
|  | appetite symptoms | PHD9-、PHD10-、PHD11-、PHD12- |
|  | independence | PHD13、PHD14、PHD15、PHD16 |
| Psychological domain | anxiety | PSD1-、PSD2-、PSD3-、PSD4-、PSD5-、PSD6-、PSD7-、PSD8- |
|  | depression | PSD9-、PSD10-、PSD11-、PSD12-、PSD13-、PSD14- |
|  | fear | PSD15-、PSD16-、PSD17- |
|  | paranoia | PSD18-、PSD19-、PSD20-、PSD21- |
| Social domain | social support | SOD1、SOD2、SOD3、SOD4、SOD5 |
|  | support utilization | SOD6、SOD7、SOD8 |
| Therapeutic domain | compliance | TRE1、TRE2 |
|  | satisfaction | TRE3、TRE4、TRE5、TRE6、TRE7、TRE8、TRE9、TRE10 |
|  | side effects of drugs | TRE11、TRE12- |
